# Popliteal pterygium syndrome

**DOI:** 10.4103/0971-9261.71747

**Published:** 2010

**Authors:** Santosh Kumar Mahalik, Prema Menon

**Affiliations:** Department of Pediatric Surgery, Advanced Pediatrics Center, Post Graduate Institute of Medical Education and Research, Chandigarh - 160 012, India

**Figure 1:**
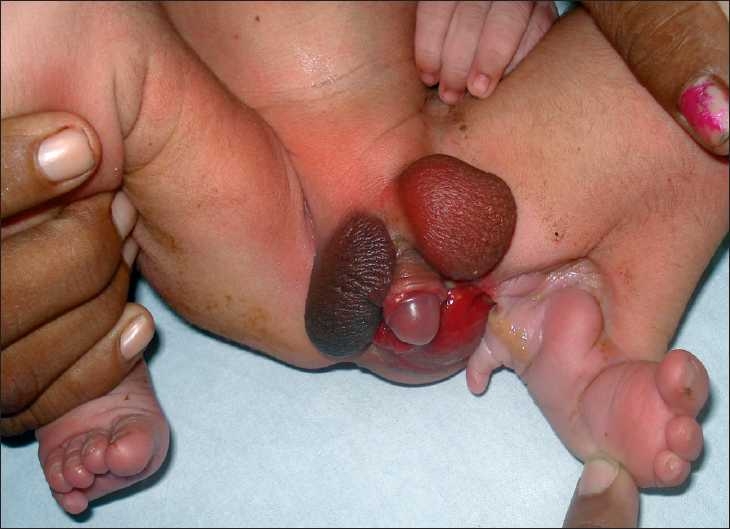
Left popliteal web with bifid scrotum, penoscrotal transposition, abnormal perineal mucosal patch, and foot anomaly

A term male baby weighing 2490 gms was seen with a rare anomaly. The left knee was flexed secondary to a tight popliteal web. The left foot had talipes equinovarus deformity with absence of 4^th^ and 5^th^ toes. Another toe was present behind the heel giving the appearance of a partial duplication of the foot. Penoscrotal transposition, bifid scrotum with bilateral descended testis, and proximal penile hypospadias were noted. A large mucosal patch was seen in the perineum on the left side [Figure 1]. The anal opening was normally located. The left kidney was not visualized on ultrasonography. Radiographs of the left lower limb showed normal long bones and a rotated calcaneum.

The popliteal pterygium syndrome is a rare autosomal dominant disorder seen in 1 in 300,000 live births. The characteristic feature is a web which usually extends from the heel to the ischial tuberosity. This contains a palpable cord of connective tissue and occasionally the popliteal artery and peroneal nerve. Absence of muscles or abnormal muscle and tendon insertion may be associated. Other anomalies show a wide range of expressivity and affect the face, limbs, and genitalia. Orofacial anomalies include cleft palate, cleft lip, micrognathia, ankyloblepharon, and choanal atresia. Anomalies of nail and digits, talipes equinovarus, spina bifida occulta, bifid ribs, and short sternum are described. Genital anomalies include hypoplastic labia majora, vagina and uterus, clitoral hypertrophy, cryptorchidism, bifid or absent scrotum and ambiguous genitalia. There is no growth disturbance and intelligence is usually normal.[1,2]

Prenatal sonography may detect an associated cleft lip/ palate along with inability of the fetus to stretch the knee. Magnetic resonance imaging is the test of choice before resection of fibrous bands and Z-plasty of the web.[3,4] Nerve grafting may be required for a short sciatic nerve. The overall prognosis is good. The index patient is awaiting staged reconstruction of the various anomalies.
